# Energy Conservation and Carbon Flux Distribution During Fermentation of CO or H_2_/CO_2_ by *Clostridium ljungdahlii*

**DOI:** 10.3389/fmicb.2020.00416

**Published:** 2020-03-17

**Authors:** Hai-Feng Zhu, Zi-Yong Liu, Xia Zhou, Ji-Hong Yi, Zeng-Min Lun, Shu-Ning Wang, Wen-Zhu Tang, Fu-Li Li

**Affiliations:** ^1^School of Biological Engineering, Dalian Polytechnic University, Dalian, China; ^2^Shandong Provincial Key Laboratory of Synthetic Biology, Key Laboratory of Biofuels, Qingdao Institute of Bioenergy and Bioprocess Technology, Chinese Academy of Sciences, Qingdao, China; ^3^State Key Laboratory of Shale Oil and Gas Enrichment Mechanisms and Effective Development, SINOPEC Exploration and Production Research Institute, Beijing, China; ^4^State Key Laboratory of Microbial Technology, Shandong University, Qingdao, China

**Keywords:** gas fermentation, *Clostridium ljungdahlii*, acetogen, biofuel, ethanol, energy conservation

## Abstract

Both CO and H_2_ can be utilized as energy sources during the autotrophic growth of *Clostridium ljungdahlii*. In principle, CO is a more energetically and thermodynamically favorable energy source for gas fermentation in comparison to H_2_. Therefore, metabolism may vary during growth under different energy sources. In this study, *C. ljungdahlii* was fed with CO and/or CO_2_/H_2_ at pH 6.0 with a gas pressure of 0.1 MPa. *C. ljungdahlii* primarily produced acetate in the presence of H_2_ as an energy source, but produced alcohols with CO as an energy source under the same fermentation conditions. A key enzyme activity assay, metabolic flux analysis, and comparative transcriptomics were performed for investigating the response mechanism of *C. ljungdahlii* under different energy sources. A CO dehydrogenase and an aldehyde:ferredoxin oxidoreductase were found to play important roles in CO utilization and alcohol production. Based on these findings, novel metabolic schemes are proposed for *C. ljungdahlii* growing on CO and/or CO_2_/H_2_. These schemes indicate that more ATP is produced during CO-fermentation than during H_2_-fermentation, leading to increased alcohol production.

## Introduction

*Clostridium ljungdahlii*, a close relative of “*Clostridium autoethanogenum*,” is used as a model organism for studying the production of ethanol and acetate from syngas, which is a gas mixture mainly composed of carbon monoxide (CO), carbon dioxide (CO_2_), and hydrogen (H_2_) (Köpke et al., 2010, 2011a; Adamberg et al., 2015; Dürre and Eikmanns, 2015; Liew et al., 2016a). Both CO and H_2_ can act as energy sources for growth and metabolism of *C. ljungdahlii* during gas fermentation. Notably, CO and H_2_ have different patterns of providing energy equivalents during metabolism. In CO-fermentation, reduced ferredoxin (Fd_red_), which is formed during CO oxidization by CO dehydrogenase, is the sole redox carrier that could allow the generation of a proton gradient across the membrane for energy conservation. On the other hand, Fd_red_ and NADPH are generated by an electron bifurcation reaction in the H_2_-fermentation by the NADP-specific [FeFe]-hydrogenase complex (Wang et al., 2013; Schuchmann and Müller, 2014; Mock et al., 2015). In addition, the free standard enthalpy changes are different for the conversion of CO or H_2_/CO_2_ to acetate and ethanol synthesis (Table 1; Wang et al., 2013; Mock et al., 2015; Esquivel-Elizondo et al., 2017). Therefore, the basic differences of energy conservation between CO and H_2_ as energy sources reveal that fermentation profiles and products are distinct in gas fermentation of *C. ljungdahlii* (Bertsch and Müller, 2015; Valgepea et al., 2018).

**TABLE 1:** Stoichiometries and free standard enthalpies of acetate and ethanol formation from CO and H_2_/CO_2_.

	**Reactions**	**ΔG°′(kJ)^a^**
1	4 CO + 2 H_2_O → CH_3_COO^–^ + H^+^ + 2 CO_2_	−175
2	4 H_2_ + 2 CO_2_ → CH_3_COO^–^ + H^+^ + 2 H_2_O	−95
3	6 CO + 3 H_2_O → CH_3_CH_2_OH + 4 CO_2_	−224
4	6 H_2_ + 2 CO_2_ → CH_3_CH_2_OH + 3 H_2_O	−105
5	11 CO + 5 H_2_O → CH_3_CHOHCHOHCH_3_ + 7 CO_2_	−388
6	1 CO + 1 Fd_ox_ + 1 H_2_O → 2 H^+^ + 1 CO_2_ + 1 Fd_red_	−14
7	2 H^+^ + 1 Fd_red_ → 1 H_2_ + 1 Fd_ox_	−7

*^*a*^Calculated from free energies of formation in reaction equations 1–7.*

**FIGURE 1:**
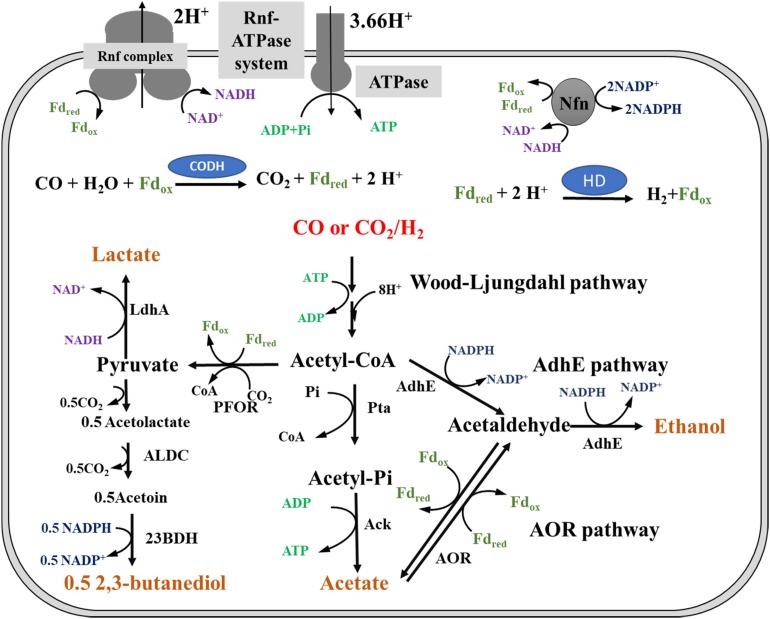
Metabolic pathway of ethanol biosynthesis in *Clostridium ljungdahlii*. Pta, phosphotransacetylase; Ack, acetate kinase; AdhE, aldehyde/alcohol dehydrogenase; AOR, acetaldehyde:ferredoxin oxidoreductase; PFOR, pyruvate:ferredoxin oxidoreductase; LdhA, lactate dehydrogenase; ALDC, acetolactate decarboxylase; 23BDH, 2,3-butanediol dehydrogenase; CODH, carbon monoxide dehydrogenase; HD, hydrogenase; Fd_red_, reduced ferredoxin; Fd_ox_, oxidized ferredoxin; Nfn, electron-bifurcating and ferredoxin-dependent transhydrogenase; Rnf complex, membrane-associated and energy-conserving reduced ferredoxin:NAD^+^ oxidoreductase; Rnf-ATPase system: a system of two enzyme complexes in which Rnf complex generates a proton gradient across the membrane by the oxidation of Fd_red_ with NAD^+^; ATPase complex, consumes the proton gradient and phosphorylates ADP to ATP in the cytoplasm; AdhE pathway, ethanol formation by AdhE catalysis; AOR pathway, AOR participates in acetate and ethanol formation.

Regardless of whether CO or H_2_ is used as an energy source, *C. ljungdahlii* must gain ATP during autotrophic growth. Moreover, *C. ljungdahlii* grows better in CO than in H_2_/CO_2_, indicating that different energy sources result in different ATP formation rates. However, the metabolic schemes of ATP generation and redox balance for cell growth and products formation by *C. ljungdahlii* growing on CO or H_2_/CO_2_ are not well understood (Schuchmann and Müller, 2014; Jones et al., 2016; Liew et al., 2016b; Valgepea et al., 2017). It has been reported that ATP formation in *C. ljungdahlii* gas fermentation relies on a Rnf-ATPase system, which can establish a proton (H^+^)-dependent transmembrane ion gradient during the Fd_red_ oxidation reaction (Figure 1; Fast and Papoutsakis, 2012; Schuchmann and Müller, 2014). It is clear that this energy conservation system is affected by the pH of the broth. The optimal pH for the growth of *C. ljungdahlii* is pH 6, which indicates that the optimal pH of the Rnf-ATPase system for ATP formation is also pH 6 (Köpke et al., 2010; Tremblay et al., 2013). Thus, the pH of the whole fermentation process was controlled at the optimal pH during the investigation of metabolic differences in CO- and H_2_-fermentation in this study.

The composition of syngas can affect the titers and ratios of acetate and ethanol, which are the major products of gas fermentation (Mohammadi et al., 2012; Aklujkar et al., 2017; Liew et al., 2017). Furthermore, 2,3-butanediol is not detected in broth during continuous fermentation by “*C. autoethanogenum*” grown on H_2_/CO_2_, but it is produced when CO is utilized as the energy source under the same growth conditions (Wang et al., 2013; Mock et al., 2015). Analysis of the metabolic pathways of ethanol and acetate indicates that acetate formation can produce ATP, whereas ethanol formation requires NADPH as a cofactor (Figure 1) during gas fermentation of *C. ljungdahlii* (Wang et al., 2013; Mock et al., 2015; Xie et al., 2015). Furthermore, acetate can be converted to ethanol through the aldehyde:ferredoxin oxidoreductase (AOR) pathway, in which ferredoxin is the essential cofactor (Liew et al., 2017). This indicates that acetate and ethanol production and their ratios in the broth are regulated by energy metabolic balance. However, the response of *C. ljungdahlii* toward different energy sources (CO or H_2_) and regulation of product formation (ethanol/acetate ratios) to maintain redox balance during autotrophic growth is still not completely understood (Richter et al., 2016; Valgepea et al., 2017).

In this study, *C. ljungdahlii* was cultured with CO:CO_2_ (80:20) or H_2_:CO_2_ (60:40) to investigate the effects of different energy sources on carbon flux distribution during autotrophic growth. The pH was controlled at pH 6 to provide constant optimal conditions for the Rnf-ATPase system. The gas pressure was controlled at 0.1 MPa to enhance gas (CO and H_2_) availability in gas-liquid fermentation bioreactor. We compared growth, product profiles, transcriptomes, and key enzyme activities of cells grown with these energy sources. We investigated the metabolic redox balance and propose metabolic schemes.

## Materials and Methods

### Bacterial Strains and Media

*Escherichia coli* strains were cultivated at 37°C in LB medium in the presence of appropriate antibiotic for general plasmid propagation and cultivation. *C. ljungdahlii* DSM 13528 was purchased from the Deutsche Sammlung von Mikroorganismen und Zellkulturen GmbH, Braunschweig, Germany and conserved by freezing mid-exponential phase cultures at −80°C with 30% glycerol. *C. ljungdahlii* was cultivated at 37°C under anaerobic conditions. A modified DSMZ 879 medium with a headspace of gas mixture (CO: CO_2_, 80:20 or H_2_: CO_2_, 60:40) as the carbon and energy source was used in gas fermentation (Xie et al., 2015). The modified DSMZ 879 medium with the following composition (per liter): 1.0 g NH_4_Cl, 0.1 g KCl, 0.2 g MgSO_4_ × 7 H_2_O, 0.8 g NaCl, 0.02 g CaCl_2_ × 2 H_2_O, 0.1 g KH_2_PO_4_, 2.5 mg Na_2_WO_4_ × 2 H_2_O, 1.0 g NaHCO_3_, 1.0 g cysteine-HCl × H_2_O, 1 g yeast extract, 0.5 g cysteine, 0.5 mg resazurin, 10 ml trace element solution and 10 ml vitamin solution. Trace element solution contains 2.0 g nitrilotriacetic acid, 1.3 g MnCl_2_ × H_2_O, 0.4 g FeSO_4_ × 7 H_2_O, 0.2 g CoCl_2_ × 7 H_2_O, 0.2 g ZnSO_4_ × 7 H_2_O, 0.2 g Na_2_MoO_4_ × 2 H_2_O, 0.02 g NiCl_2_ × 6 H_2_O and 0.1 g Na_2_SeO_3_ × 5 H_2_O in 1 L distilled water. Vitamin solution involves 2 mg biotin, 2 mg folic acid, 10 mg pyridoxine–HCl, 25 mg thiamine-HCl × 2 H_2_O, 5 mg riboflavin, 5 mg Nicotinic acid, 5 mg D-Ca-pantothenate, 0.1 mg vitamin B12, 5 mg ρ-aminobenzoic acid and 5 mg lipoic acid in 1 L distilled water. Analytical grade chemicals used in the medium were purchased from Sinopharm Chemical Reagent Co., Ltd. (Shanghai, China). All antibiotics were purchased from Sangon Co., Ltd. (Shanghai, China).

### Fed-Batch Fermentation With Syngas

Batch fermentation was performed in a 250-ml screw-cap bottle with a 50-ml working volume of modified DSMZ 879 medium as pre-culture. The medium was assembled in anaerobic chamber (COY Laboratory Products, Grass Lake, MI, United States). After autoclaving, FeSO_4_, vitamins, cysteine-HCl and NaHCO_3_ were added using syringe with 0.2 μm filter. Then gas in the headspace was substituted by syngas as required with a pressure of 0.2 MPa. Fed-batch fermentation with pH control was carried out in a FUS-5L bioreactor in duplicate (Guoqiang Biotech Co. Ltd., Shanghai, China) containing 2.5 L of modified DSM 897 medium. The supplied gas pressure was controlled at 0.1 MPa and the gas flow rate was 30 ml/min during the whole fermentation process in the bioreactor. Bioreactor pH was controlled at 6 automatically by adding 4 M KOH. 300 mL pre-culture of *C. ljungdahlii* was inoculated into the bioreactor and 5 mL samples were withdrawn every 12 h for cell density monitoring and products analysis.

### Gene Expression Analysis by RNA-Seq

Comparative transcriptomics of cells grown on CO and H_2_/CO_2_ was performed to investigate gene expression profiles based on three biological replicates. Cell pellets from cultures in the bioreactor were collected by centrifugation at 10000 × *g* under −4°C for 10 min at exponential phase and frozen in liquid nitrogen immediately and stored at −80°C. The RNA isolation and high-through RNA sequencing (RNA-Seq) were accomplished by Allwegenetech Corp (Beijing, China). Total RNA was extracted using the mirVana miRNA Isolation Kit (Ambion, Santa Clara, CA, United States) following the manufacturer’s protocol. RNA integrity was evaluated using the Agilent 2100 Bio-analyzer (Agilent Technologies, Santa Clara, CA, United States). The samples with RNA Integrity Number (RIN) ≥ 7 were subjected to subsequent analysis. The libraries were constructed using TruSeq Stranded mRNA LTSample Prep Kit (Illumina, San Diego, CA, United States) according to the manufacturer’s instructions. Then these libraries were sequenced on the Illumina sequencing platform (HiSeqTM 2500) and 150 bp/125bp paired-end reads were generated. Based on reads per kilobase of transcript per million mapped reads (RPKM) normalization, the genes expression profiles were analyzed. The processed RNA-Seq data were submitted to the ArrayExpress database^1^ under the accession number E-MEAB-8260.

### Preparation of Cell Extracts and Enzyme Activity Analysis

500 mL exponential cells growing on CO or H_2_/CO_2_ were collected by centrifugation at 10000 × *g* under 4°C under strictly anoxic condition. The pellets were suspended in 20 mL of anoxic 50 mM potassium phosphate (pH 7.4), containing 2 mM DTT. Lysozyme was added to the cell suspension before incubation at 37°C for 30 min. Then the mixture was moved to anaerobic chamber for ultrasonication. Finally, cells debris was removed by centrifugation at 35000 × *g* and 4°C for 1 h. The supernatant was transferred to a new tube for enzyme assay and protein concentration determination using Bio-Rad protein assay with bovine serum albumin as the standard (Wang et al., 2013).

Acetaldehyde:ferredoxin oxidoreductase (AOR) activities were determined under strictly anoxic condition at 37°C in 1.5-mL anaerobic cuvettes sealed with rubber stopper (Hellma GmbH, Müllheim, Germany). The cuvettes were filled with pure N_2_ at 1.2 × 10^5^ Pa as the gas phase before use to maintain anaerobic condition during enzyme catalysis. The reactions were monitored photometrically at the specified wavelength. Ferredoxin reduction was monitored at 430 nm (Δε_*ox–red*_≈13.1 mM^–1^cm^–1^). One unit (1 U) was defined as the transfer of 2 μmol electrons min^–1^. The assay mixture contained 50 mM Tris–HCl (pH 7.4), 2 mM DTT, 1.5 mM acetaldehyde, and 30 μM ferredoxin in the AOR activity assay (Wang et al., 2013).

Ferredoxin of “*C. autoethanogenum*” (WP_013236834.1) was obtained by heterologous expression in *E. coli*. Gene amplification was performed by PCR with genomic DNA of “*C. autoethanogenum*” as the template. The following primers were used: 5′-CATGCCATGGCATATAAAATTACAGAGGAT-3′ (reverse primer, the *Nco*I restriction site is underlined); 5′-CCGCTCGAGGCTTTCTTCAACTGGTGCTC-3′ (forward primer, the *Xho*I restriction site is underlined). The PCR fragment was digested by restriction endonucleases and subsequently ligated into expression vector *p*ET28b, which had been digested by the same restriction endonucleases. Finally, the constructed plasmid was transformed into *E. coli* C41 (DE3), which already harbored plasmids *p*RKISC and *p*CodonPlus for production of iron-sulfur proteins. The cell cultivation and ferredoxin purification steps were performed according to the previous description (Demmer et al., 2015). Ferredoxin was stored at −20°C in an N_2_ atmosphere until use.

### Analytical Methods

The concentrations of ethanol, acetic acid, lactate, and 2,3-butanediol were determined using an Agilent 1100 high-performance liquid chromatography (HPLC) system with an Agilent Hi-Plex H column (Agilent Technologies, Santa Clara, CA, United States) equipped with a refractive index detector operated at 35°C. Column temperature was maintained at 55°C. Slightly acidified (5 mM H_2_SO_4_) water was used as the mobile phase at a flow rate of 0.7 ml/min.

The cells growing on gas under controlled pH and gas pressure were withdrawn from the bioreactor at 12 h interval. The growth of *C. ljungdahlii* was monitored by using a 2600 spectrophotometer (Unico instrument company, China) to measure the optical densities at 600 nm in a quartz-type cuvette (Hellma GmbH, Müllheim, Germany).

## Results and Discussion

### Fed-Batch Fermentation in the Case of pH and Gas Pressure Control

**FIGURE 2:**
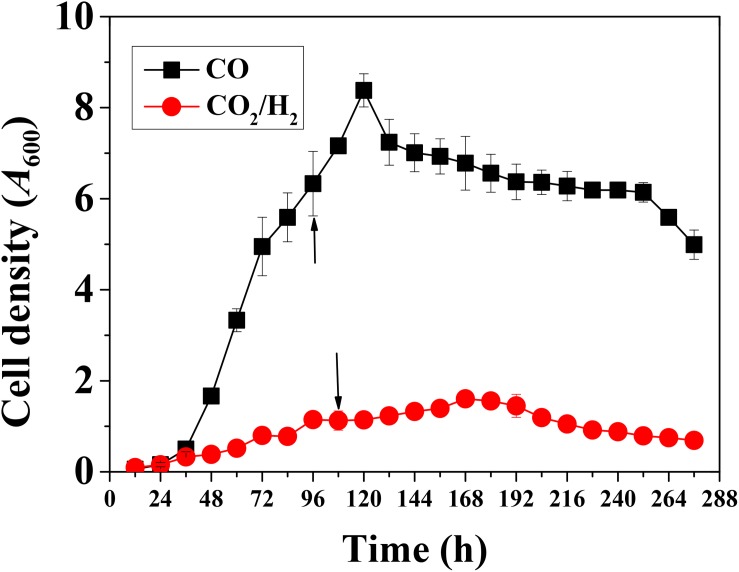
Cell growth of *Clostridium ljungdahlii* with CO or H_2_/CO_2_. Arrows: These two time points represent exponential growth phases, respectively. Samples were withdrawn from fermenter at these time points for RNA-Seq analysis.

*Clostridium ljungdahlii* grew well in the case of gas fermentation with CO/CO_2_ as carbon and energy source and the optical density (OD, 600 nm) reached 8.4 ± 0.4 after 120 h. In contrast, the strain grew poorly in the presence of H_2_/CO_2_ as carbon and energy source with peak cell density of 1.6 ± 0.3 after 168 h (Figure 2). These results indicate that *C. ljungdahlii* can more easily gain energy from CO than from H_2_ for autotrophic growth under the same fermentation conditions.

**FIGURE 3:**
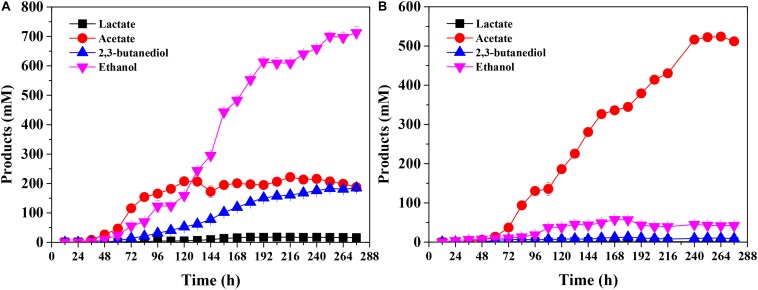
Product concentration of *Clostridium ljungdahlii* grown on CO **(A)** and H_2_/CO_2_ **(B)** at pH 6.0 and gas pressure of 0.1 MPa.

Regarding the products, *C. ljungdahlii* mainly produced ethanol (713 ± 21 mM) in the presence of CO as energy source in the end-products. Furthermore, the concentrations of 2,3-butanediol and acetate were 188 ± 4 mM and 185 ± 7 mM, respectively (Figure 3A). On the other hand, acetate was found to be dominant among the end-products in the presence of H_2_ as energy source, achieving 512 ± 6 mM at the end of the experiment (Figure 3B). These results clearly show that different energy sources affect not only biomass accumulation but also product titers in fed-batch fermentation of *C. ljungdahlii*.

ATP formation is highly susceptible to pH changes in *C. ljungdahlii* gas fermentation (Figure 1; Schuchmann and Müller, 2014). Furthermore, gas-liquid mass transfer limitation results in the inefficient utilization of CO or H_2_/CO_2_ (Ungerman and Heindel, 2007; Xu et al., 2017). As a result, *C. ljungdahlii* produces low biomass and low ethanol and acetate titers in the traditional batch gas fermentation (Ungerman and Heindel, 2007; Köpke et al., 2011b; Xie et al., 2015; Xu et al., 2017). In this study, we improved the fermentation conditions and provided ideal growth conditions for *C. ljungdahlii* via pH and gas pressure control during autotrophic growth (Figure 3). *C. ljungdahlii* exhibited distinct differences in fermentation profiles when grown on CO and/or H_2_/CO_2_. This information is useful for studying differences in energy metabolism when acetogenic bacteria grown on different energy sources.

### Genome-Wide Transcriptional Analysis With CO or H_2_ as Energy Source

Comparative transcriptomics was conducted by RNA-Seq technology for the investigation of the intracellular flux patterns at the transcriptional levels. The original and processed RNA-Seq data were submitted to the ArrayExpress database^1^ under accession number E-MEAB-8260. Supplementary Table S1 shows the expression profiles of 62 genes located in the central carbon and energy metabolic pathways (Köpke et al., 2010, 2011b). Among these, we particularly focused on the genes with transcriptional reads per kilobase of transcript per million mapped reads (RPKM) greater than 50 and change folds greater than 2 (log_2_ value greater than 1 or less than −1).

The energy supply modes are different for *C. ljungdahlii* during autotrophic growth on CO or H_2_. Fd_red_, formed during CO oxidation to CO_2_ by CO dehydrogenase (CODH, *cooS*), is the initial energy source in CO fermentation. Both Fd_red_ and NADPH, formed simultaneously by electron bifurcation via hydrogenase, are the initial energy sources in H_2_ fermentation (Wang et al., 2013; Mock et al., 2015). Therefore, we investigated the transcriptional levels of the CODH and hydrogenase genes. There are four putative genes/gene clusters, i.e., CLJU_c01650, CLJU_c09110, CLJU_c37560 and CLJU_c37660-70 encoding CODH, among which only the transcriptional level of CLJU_c09110 was induced during CO fermentation, in comparison with H_2_ fermentation (Supplementary Table S1; Köpke et al., 2010). Furthermore, there are four putative hydrogenases in *C. ljungdahlii*, based on genome sequence analysis. The genes CLJU_c28660-70 and CLJU_c23060-90 showed few changes in gene expression under both CO fermentation and H_2_ fermentation (Köpke et al., 2010). The gene expression level of CLJU_c37220 encoding an Fe-only hydrogenase was higher in H_2_ fermentation than in CO fermentation (Supplementary Table S1). The role of Fe-only hydrogenase is oxidation of reduced ferredoxin, and we speculate its expression was inhibited in presence of CO to some extent (Goldet et al., 2009). The fourth hydrogenase gene is located in a large gene cluster (CLJU_c06990-07080), and its expression level was higher under H_2_ fermentation than under CO fermentation (Supplementary Table S1). The function of this gene cluster has been clarified in “*C. autoethanogenum*,” which encodes a NADP-specific electron bifurcating hydrogenase in complex with formate dehydrogenase (Wang et al., 2013). Therefore, this hydrogenase plays a critical role in providing reducing equivalents in H_2_ fermentation.

**FIGURE 4:**
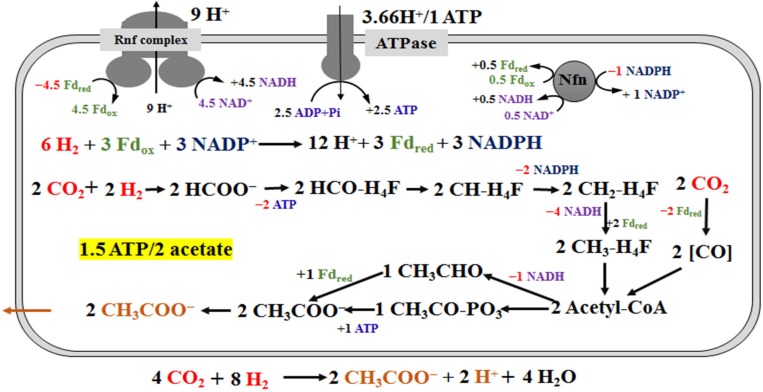
Schemes of the metabolism of *Clostridium ljungdahlii* grown on H_2_/CO_2_ at pH 6.0 and gas pressure of 0.1 MPa. For simplification, an electron-bifurcating methylene-THF reductase is assumed here, and protons in the individual reactions are omitted. The energy source (H_2_) and carbon source (CO_2_) are in red and the product (acetate) is in orange. “+ ×” indicates ATP and reduced electron carriers (Fd_red_, NADH and NADPH), which are in different color in the scheme, are produced. On the contrary, “**−** ×” indicates ATP and reduced electron carriers are consumed in redox reactions.

The product concentrations were remarkably different for CO fermentation and H_2_ fermentation (Figure 3). The related genes for product biosynthesis were also analyzed (Figure 1; Köpke et al., 2010). Comparative transcriptomics data showed that the expression level of 2,3-butanediol dehydrogenase, which was encoded by CLJU_c01650, was higher in the CO fermentation than that in the H_2_ fermentation (Supplementary Table S1; Tan et al., 2015), and these transcriptome results were consistent with those of the 2,3-butanediol fermentation titer (Figure 3). However, the expression levels of genes involved in acetate and ethanol formation were lower under CO fermentation (Figure 1; Köpke et al., 2010). Of note, the RPKM values of an AOR gene encoded by CLJU_c20210 and a pyruvate:ferredoxin oxidoreductase (PFOR) gene encoded by CLJU_c09340 were high in both the CO and H_2_ fermentation. This indicates these two functional enzymes play crucial roles during gas fermentation. It has been reported that ethanol formation is mainly dependent on the AOR pathway during gas fermentation (Mock et al., 2015; Liew et al., 2017). Our results are consistent with the finding that the *aor2* gene is strongly expressed during autotrophic growth in CO in previous studies. Interestingly, *aor2* was also transcribed at a high level when grown with H_2_/CO_2_, suggesting that AOR is also active in H_2_ fermentation (Supplementary Table S1). The specific activity of acetaldehyde:ferredoxin oxidoreductase was determined in the cell extracts growing on CO (6.7 U/mg) and H_2_/CO_2_ (2.5 U/mg). This result also shows that AOR is functional during H_2_/CO_2_ fermentation. However, the ethanol titer (42 ± 1 mM) was very low under these fermentation conditions (Figure 3B). This can be elucidated by the fact that partial acetate in the broth comes from the oxidation of acetaldehyde (Figures 1, 4). This result indicates that AOR (CLJU_c20210) catalyzed the reaction from acetate to acetaldehyde in CO fermentation, but catalyzed the inverse reaction in H_2_ fermentation. We suggest that this flexible mechanism aids in maintaining redox balance in response to different fermentation conditions.

The low biomass accumulation indicated that ATP supply was low during growth with H_2_. Thus, the genes associated with ATP formation, including Rnf–ATPase genes and *nfn*, had higher transcriptional levels (Supplementary Table S1). It is clear that low levels of ATP not only reduced biomass but also decreased alcohol production in this study and previous reports (Valgepea et al., 2018).

### Calculation of ATP Gains During CO and H_2_ Fermentation

Mock et al. (2015) completed a metabolic scheme for “*C. autoethanogenum*” in H_2_/CO_2_ fermentation. We fully agree with the principles of metabolic pathways and energy conservation in this scheme, but modified the pathway of acetate synthesis. The deletion of the acetate formation pathway through the inactivation of phosphate acetyltransferase, encoded by *pta* (CLJU_c12770), leads to lethal in gas fermentation (Huang et al., 2016). Therefore, the acetate biosynthesis pathway from acetyl-CoA is necessary in the scheme. Meanwhile, acetate formation from acetaldehyde should also be included, based on AOR specific activity (2.5 U/mg) verified in this study and transcriptomics data (Supplementary Table S1). Furthermore, we cannot rule out the possibility, that H_2_ was produced during the fermentation process, yet H_2_ concentrations were not monitored in this study. The scheme of energy metabolism of *C. ljungdahlii* is given in Figure 4, under the assumption that only acetate is formed in H_2_/CO_2_ fermentation (Figure 3B). Our metabolic scheme indicates that 0.75 mole ATP is produced during 1 mole of acetate formed from H_2_/CO_2_ (Figure 4).

**FIGURE 5:**
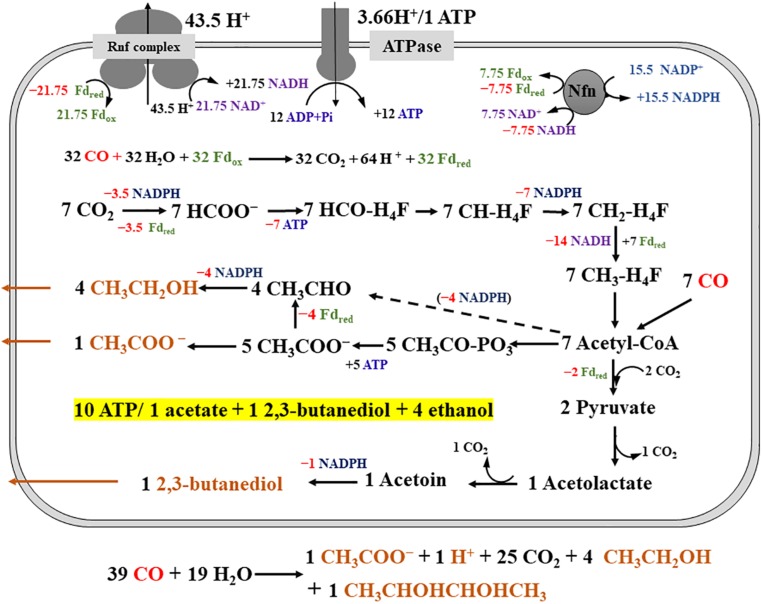
Schemes of the metabolism of *Clostridium ljungdahlii* grown on CO at pH 6.0 and gas pressure 0.1 MPa. For simplification, an electron-bifurcating methylene-THF reductase is assumed, ethanol is produced via acetic reduction to acetaldehyde, and protons in the individual reactions are omitted. The energy and carbon source (CO) is in red and the products (acetate, ethanol, and 2,3-butanediol) are in orange. “+ ×” indicates ATP and reduced electron carriers (Fd_red_, NADH and NADPH), which are in different color in the scheme, are produced. On the contrary, “**−**×” indicates ATP and reduced electron carriers are consumed in redox reactions. The dashed arrow means the redox reaction is also a possible pathway for ethanol production.

*Clostridium ljungdahlii* exhibited a significant difference in alcohol production in CO fermentation as compared with that in H_2_/CO_2_ fermentation. Ethanol was the main product in CO fermentation (Figure 3A), suggesting a key role of AOR, converting acetate to acetaldehyde for further reduction to ethanol by AdhE in the metabolism of CO (Figure 1). Gene knockout studies in “*C. autoethanogenum*” demonstrated AOR is critical to ethanol formation (Liew et al., 2017). Based on these findings and equations in the Table 1, three schemes of the energy metabolism of *C. ljungdahlii* are exhibited under three assumptions: (i) only acetate formation (Supplementary Figure S1); (ii) acetate and 2,3-butanediol formed (Supplementary Figure S2); and (iii) acetate, ethanol, and 2,3-butanediol formed (Figure 5). Among these three schemes, Figure 5 most closely reflects the actual metabolic process in CO fermentation found in this study. This scheme indicates that 10 moles ATPs are produced during formation of 1 mole of acetate, 1 mole of 2,3-butanediol, and 4 moles of ethanol from CO. The mole ratio of dominant end-products (acetate, 2,3-butanediol, and ethanol) is very close to 1:1:4 (Figure 3A).

All of the acetogenic bacteria are able to produce acetate via Wood-Ljungdahl pathway during CO and/or H_2_/CO_2_ fermentation (Schuchmann and Müller, 2014). However, only some acetogenic bacteria, including *C. ljungdahlii*, can grow in the presence of CO to produce ethanol (Köpke et al., 2011b). This indicates that *C. ljungdahlii* has a unique mechanism to achieve CO fixation and energy conservation (Buckel and Thauer, 2018; Peters et al., 2018). Based on findings in this work, we speculate that an independent and specific CODH is necessary for *C. ljungdahlii* fermentation on CO. This enzyme is used to convert CO to CO_2_ for formation of Fd_red_, which provides reducing equivalents in the fermentation (Supplementary Table S1; Köpke et al., 2010). Furthermore, the AOR pathway plays an important role in *C. ljungdahlii* gas fermentation. AOR, together with the bi-functional aldehyde/alcohol dehydrogenase (AdhE), can achieve flexible conversion between two C2-compounds, ethanol and acetate (Liew et al., 2017). Ethanol formation by the AOR pathway requires sufficient energy equivalents (NADPH and Fd_red_); on the contrary, this reaction can provide energy equivalents to support cell metabolism via ethanol oxidation (Figure 1). Therefore, the ratio between ethanol and acetate is closely associated with redox balance but not with carbon flux balance. Owing to these characteristics, *C. ljungdahlii* and “*C. autoethanogenum*” grow better, and produce more ATP and ethanol in CO than that in H_2_/CO_2_ (Mock et al., 2015; Liew et al., 2017).

The low yields of 2,3-butanediol and lactate result in poor understanding of the metabolic mechanism of these two products (Köpke et al., 2011b; Wang et al., 2013; Mock et al., 2015; Valgepea et al., 2018). Our fermentation technology increased the titer of 2,3-butanediol to 188 ± 4 mM (Figure 3). Based on our knowledge, this is the highest titer of 2,3-butanediol in gas fermentation among the published reports. Importantly, these results provide a platform to study the biosynthesis and metabolism of 2,3-butanediol in the future.

## Conclusion

*Clostridium ljungdahlii* is able to produce ethanol and acetate with CO as the carbon and energy source, unlike other acetogenic bacteria with acetate as the main product. To elucidate this unique metabolism, we cultivated *C. ljungdahlii* with CO or H_2_/CO_2_ using a fed-batch fermentation technology with pH and gas pressure control. The results show that *C. ljungdahlii* mainly produced alcohols (ethanol and 2,3-butanediol) under CO fermentation and mainly produced acetate under H_2_/CO_2_ fermentation. The comparative transcriptomics analysis and AOR activities suggest that a CODH (encoded by CLJU_09110) and an AOR (encoded by CLJU_20210) play important roles in CO metabolism. This CODH can provide an energy equivalent (Fd_red_), as required, by oxidizing CO to CO_2_ for metabolism in CO fermentation. Additionally, the AOR pathway can provide a flexible regulation mechanism for energy balance by the conversion of acetate and ethanol. According to these results and previous reports, we propose metabolic schemes for *C. ljungdahlii* growing on CO and/or H_2_/CO_2_. Stoichiometric analysis of ATP gains estimated that ATP yield is 0.75 ATP with 1 mole of acetate formed during autotrophic growth on H_2_/CO_2_, in contrast to 10 moles of ATPs with 1 mole of acetate, 1 mole of 2,3-butanediol, and 4 moles of ethanol formed in *C. ljungdahlii* fermentation on CO at pH 6.0.

## Data Availability Statement

The datasets generated for this study can be found in the ArrayExpress database (www.ebi.ac.uk/arrayexpress) under the accession number E-MEAB-8260.

## Author Contributions

F-LL and W-ZT conceived and designed the study. H-FZ, Z-YL, and J-HY performed the experiments. S-NW analyzed the enzyme activities data. Z-YL completed metabolic schemes guided by F-LL. Z-YL and F-LL wrote the manuscript with input from all authors.

## Conflict of Interest

The authors declare that the research was conducted in the absence of any commercial or financial relationships that could be construed as a potential conflict of interest.

## Footnotes

1 www.ebi.ac.uk/arrayexpress
