# Supplementary material for: Energy Conservation and Carbon Flux Distribution During Fermentation of CO or H2/CO2 by Clostridium ljungdahlii
Source: Front Microbiol. 2020 Mar 17;11:416. doi: 10.3389/fmicb.2020.00416 (PMC7092622; doi:10.3389/fmicb.2020.00416)
Supplement: Supplementary file 1 [file Data_Sheet_1.docx]

Supplemental Materials for

**Energy Conservation and Carbon Flux Distribution during Fermentation of CO or H_2_/CO_2_ by *Clostridium ljungdahlii***

*Hai-Feng Zhu^1#^, Zi-Yong Liu^2#^, Xia Zhou^3^, Ji-Hong Yi^4^, Zeng-Min Lun^3^, Shu-Ning Wang^4^, Wen-Zhu Tang^1^*, Fu-Li Li^2^**

^1^School of Biological Engineering, Dalian Polytechnic University, Dalian 116034, P. R. China;

^2^Shandong Provincial Key Laboratory of Synthetic Biology, Key Laboratory of Biofuels, Qingdao Institute of Bioenergy and Bioprocess Technology, Chinese Academy of Sciences, 266101 Qingdao, P. R. China;

^3^State Key Laboratory of Shale Oil and Gas Enrichment Mechanisms and Effective Development, SINOPCE Exploration & Production Research Institute, 100083 Beijing, China

^4^State Key Laboratory of Microbial Technology, Shandong University, Qingdao, 266237, P. R. China;

*^#^*The authors contributed equally to this work

*Corresponding author:

Fu-Li Li, Phone: +86 532-80662655; Fax: +86 532-80662778; Email: lifl@qibebt.ac.cn. Postal address: No. 189 Songling Rd., Qingdao 266101, P. R. China

Wen-Zhu Tang, Phone: +86 411-86323646; Fax: +86 411-86323646; Email: tangwenzhu2000@163.com. Postal address: No. 1 Qinggongyuan, Ganjingzi, Dalian 116034, P. R. China

**Table S1 The expression profiles of the genes located in the central metabolic pathways during fermentation grown on CO and CO_2_/H_2_ of *Clostridium ljungdahlii*.**

| **ORF** | **Gene and Protein** | **RPKM^b^** | | |  |
| --- | --- | --- | --- | --- | --- |
|  |  | CO  *t* =96 h | | CO_2_/H_2_  *t* =108 h | Change fold^a^ |
| **Wood-Ljungdahl pathway genes** | | | | | |
| CLJU_RS04490/ CLJU_c09110 | *cooS*, carbon-monoxide dehydrogenase catalytic subunit | 6553 | 2834 | | **1.2** |
| CLJU_RS08800/ CLJU_c01650 | *cooS*, carbon-monoxide dehydrogenase catalytic subunit | 9 | 12 | | **-0.5** |
| CLJU_RS18325/ CLJU_c37220 | *hyd*, 4Fe-4S dicluster domain-containing protein | 936 | 1221 | | **-0.4** |
| CLJU_RS14140/ CLJU_c28660 | *hyd*, Ni/Fe hydrogenase | 0 | 0 | | **0** |
| CLJU_RS14145/ CLJU_c28670 | *hyd*, Ni/Fe hydrogenase | 0 | 0 | | **0** |
| CLJU_RS11345/ CLJU_c23060 | *hypE*, hydrogenase expression/formation protein | 46 | 55 | | **-0.3** |
| CLJU_RS11350/ CLJU_c23070 | *hypD*, hydrogenase formation protein | 50 | 56 | | **-0.2** |
| CLJU_RS11355/ CLJU_c23080 | HupF family hydrogenase formation chaperone | 0 | 0 | | **0** |
| CLJU_RS11360/ CLJU_c23090 | *hypF*, carbamoyltransferase | 77 | 84 | | **-0.1** |
| CLJU_RS18490/ CLJU_c37550 | *cdhC*, CO dehydrogenase/CO-methylating acetyl-CoA synthase complex subunit beta | 24555 | 39224 | | **-0.7** |
| CLJU_RS18495/ CLJU_c37560 | carbon monoxide dehydrogenase | 10142 | 15366 | | **-0.6** |
| CLJU_RS18500/ CLJU_c37570 | acetyl-CoA synthase /synthase complex subunit gamma | 10526 | 19160 | | **-0.9** |
| CLJU_RS18505/ CLJU_c37580 | acetyl-CoA synthase /synthase complex subunit delta | 5 | 9 | | **-0.7** |
| CLJU_RS18510/ CLJU_c37590 | carbon monoxide dehydrogenase | 6002 | 10850 | | **-0.9** |
| CLJU_RS18515/ CLJU_c37600 | *lpdA*, dihydrolipoyl dehydrogenase | 4738 | 7880 | | **-0.7** |
| CLJU_RS18520/ CLJU_c37610 | *metF*, 5,10-methylenetetrahydrofolate reductase | 15128 | 206638 | | **-0.8** |
| CLJU_RS18525/ CLJU_c37620 | hypothetical protein, 5,10-methylene-tetrahydrofolate | 7822 | 10352 | | **-0.4** |
| CLJU_RS18530/ CLJU_c37630 | *folD*, dehydrogenase/5,10-methylene-tetrahydrofolate cyclohydrolase | 5451 | 7463 | | **-0.4** |
| CLJU_RS18535/ CLJU_c37640 | sugar ABC transporter substrate-binding protein | 8302 | 11394 | | **-0.5** |
| CLJU_RS18540/ CLJU_c37650 | *fhs*, formate-tetrahydrofolate ligase | 11475 | 15965 | | **-0.5** |
| CLJU_RS18545/ CLJU_c37660 | carbon monoxide dehydrogenase | 38718 | 52819 | | **-0.4** |
| CLJU_RS18550/ CLJU_c37670 | *cooS*, carbon-monoxide dehydrogenase catalytic subunit | 3787 | 5802 | | **-0.6** |
| CLJU_RS04405/CLJU_c08930 | *fdh2*,formate dehydrogenase subunit alpha | 3723 | 2764 | | **0.4** |
| CLJU_RS09825/CLJU_c20040 | *fdh3*, formate dehydrogenase H subunit alpha | 4765 | 6814 | | **-0.5** |
| CLJU_RS03440/CLJU_c06990 | *fdh1*, formate dehydrogenase H subunit alpha | 3287 | 7616 | | **-0.9** |
| CLJU_RS03445/CLJU_c07000 | *moeA*, molybdopterin molybdenumtransferase MoeA | 510 | 667 | | **-0.1** |
| CLJU_RS03450/CLJU_c07010 | *mobB*, molybdopterin-guanine dinucleotide biosynthesis protein B | 211 | 291 | | **-0.4** |
| CLJU_RS03455/CLJU_c07020 | *fdhD*, sulfurtransferase FdhD | 543 | 895 | | **-0.6** |
| CLJU_RS03460/CLJU_c07030 | NADH-quinone oxidoreductase subunit NuoE | 2168 | 3469 | | **-0.7** |
| CLJU_RS03465/CLJU_c07040 | NADH dehydrogenase | 3251 | 4978 | | **-0.7** |
| CLJU_RS03470/CLJU_c07050 | 2Fe-2S cluster binding domain-containing protein | 1419 | 2339 | | **-0.7** |
| CLJU_RS03475/CLJU_c07060 | 4Fe-4S dicluster domain-containing protein | 2125 | 3728 | | **-0.8** |
| CLJU_RS03480/CLJU_c07070 | 4Fe-4S dicluster domain-containing protein | 3851 | 6348 | | **-0.7** |
| CLJU_RS03485/CLJU_c07080 | 4Fe-4S dicluster domain-containing protein | 1983 | 3049 | | **-0.6** |
| **Products synthesis genes** | | | | | |
| CLJU_RS08095/CLJU_c16510 | *adhE1*, bifunctional acetaldehyde-CoA/alcohol dehydrogenase | 106 | 240 | | **-1.2** |
| CLJU_RS08100/CLJU_c16520 | *adhE2*, bifunctional acetaldehyde-CoA/alcohol dehydrogenase | 4 | 2 | | **1.0** |
| CLJU_RS09865/CLJU_c20110 | *aor1*, aldehyde ferredoxin oxidoreductase | 68 | 59 | | **0.2** |
| CLJU_RS09915/CLJU_c20210 | *aor2*, aldehyde ferredoxin oxidoreductase | 24321 | 23983 | | **0.02** |
| CLJU_RS06260/CLJU_c12770 | *pta*, phosphate acetyltransferase | 881 | 1387 | | **-0.7** |
| CLJU_RS06265/CLJU_c12780 | *ack*, acetate kinase | 1024 | 1723 | | **-0.8** |
| CLJU_RS04605/CLJU_c09340 | *pfor*, pyruvate:ferredoxin oxidoreductase | 15935 | 28216 | | **-0.8** |
| CLJU_RS14475/CLJU_c29340 | *pfor*, pyruvate:ferredoxin oxidoreductase | 20 | 30 | | **-0.6** |
| CLJU_RS15865/CLJU_c32190 | *ldhA*, lactate dehydrogenase | 136 | 168 | | **-0.3** |
| CLJU_RS11425/CLJU_c23220 | *bdh*, 2,3-butanediol dehydrogenase | 18045 | 455 | | **5.3** |
| **Energy conservation metabolic pathway genes** | | | | | |
| CLJU_RS01160/CLJU_c02370 | F0F1 ATP synthase subunit A | 491 | 849 | | **-0.8** |
| CLJU_RS01165/CLJU_c02380 | *atpE*, ATP synthase F0 subunit C | 104 | 207 | | **-1.0** |
| CLJU_RS01170/CLJU_c02390 | *atpF*, ATP synthase F0 subunit B | 337 | 591 | | **-0.8** |
| CLJU_RS01175/CLJU_c02400 | F0F1 ATP synthase subunit delta | 326 | 584 | | **-0.8** |
| CLJU_RS01180/CLJU_c02410 | F0F1 ATP synthase subunit alpha | 1211 | 2399 | | **-1.0** |
| CLJU_RS01185/CLJU_c02420 | F0F1 ATP synthase subunit gamma | 592 | 1195 | | **-1.0** |
| CLJU_RS01190/CLJU_c02430 | *atpD*, F0F1 ATP synthase subunit beta | 1140 | 2327 | | **-1.0** |
| CLJU_RS01195/CLJU_c02440 | *atpC*, ATP synthase F1 subunit epsilon | 455 | 879 | | **-1.0** |
| CLJU_RS05570/CLJU_c11360 | *rnfC*, type electron transport complex subunit, Rnf C | 8769 | 10646 | | **-0.3** |
| CLJU_RS05575/CLJU_c11370 | *rnfD*, NADH:ubiquinone oxidoreductase, Rnf D | 4610 | 5733 | | **-0.3** |
| CLJU_RS05580/CLJU_c11380 | *rnfG*, FMN-binding protein, Rnf G | 2312 | 2915 | | **-0.3** |
| CLJU_RS05585/CLJU_c11390 | *rnfE*, electron transport complex subunit, Rnf E | 2247 | 2836 | | **-0.3** |
| CLJU_RS05590/CLJU_c11400 | *rnfA*, electron transport complex subunit, Rnf A | 2379 | 3010 | | **-0.3** |
| CLJU_RS05595/CLJU_c11410 | *rnfB*, 4Fe-4S dicluster domain-containing protein, RnfB | 6029 | 7553 | | **-0.3** |
| CLJU_RS18335/CLJU_c37240 | *nfn* ,glutamate synthase (NADPH)-homotetrameric. Nfn | 5259 | 17415 | | **-1.7** |

Genes are listed in order of old and new ORF numbers; genes located in the same operon are assembled by same shading; ORF: Open reading frame;

^a^:Data represent the log_2_ values change folds of PRKM during CO-fermentation, as compared to those CO_2_/H_2_-fermetation.

^b^:RPKM is the abbreviation of Reads Per Kilobase per Million mapped reads.


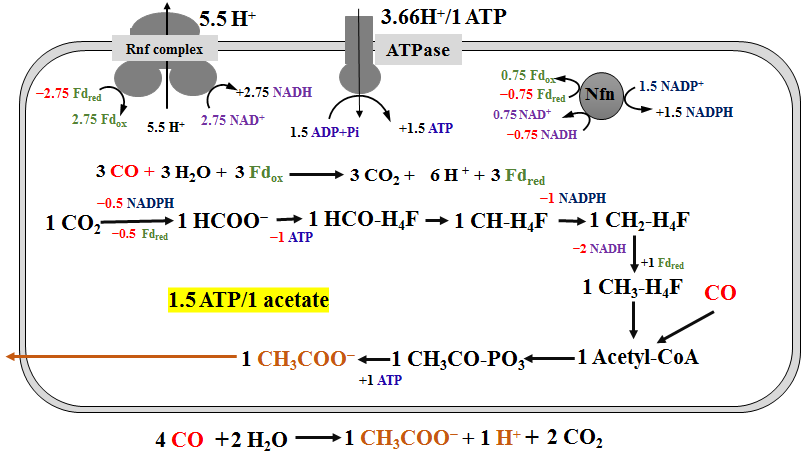


Figure S1 Schemes of the metabolism of *Clostridium ljungdahlii* grown on CO with pH 6.0 and gas pressure 0.1 MPa. For simplification, an electron-bifurcating methylene-THF reductase is assumed here, and protons in the individual reactions are omitted. The energy and carbon source (CO) are in red and the product (acetate) is in orange. “+ x” indicates ATP and reduced electron carriers (Fd_red_, NADH and NADPH), which are in different colour in the scheme, are produced. On the contrary, “**−** x” indicates ATP and reduced electron carriers are consumed in redox reactions.


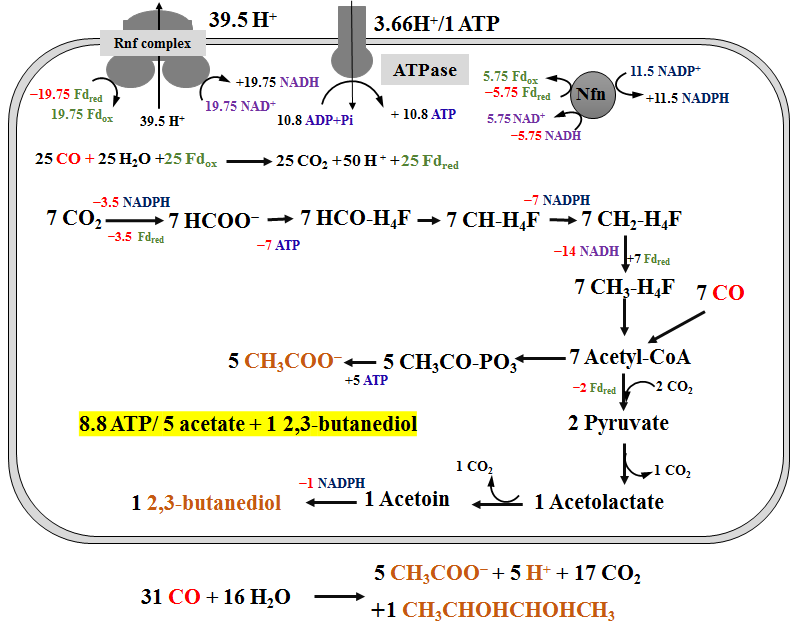


Figure S2 Schemes of the metabolism of *Clostridium ljungdahlii* grown on CO with pH 6.0 and gas pressure 0.1 MPa. For simplification, an electron-bifurcating methylene-THF reductase is assumed here, and protons in the individual reactions are omitted. The energy and carbon source (CO) is in red and the products (acetate and 2,3-butanediol) are in orange. “+ x” indicates ATP and reduced electron carriers (Fd_red_, NADH and NADPH), which are in different colour in the scheme, are produced. On the contrary, “**−** x” indicates ATP and reduced electron carriers are consumed in redox reactions.
